# Organically interconnected graphene flakes: A flexible 3-D material with tunable electronic bandgap

**DOI:** 10.1038/s41598-019-50037-y

**Published:** 2019-09-23

**Authors:** E. Klontzas, E. Tylianakis, V. Varshney, A. K. Roy, G. E. Froudakis

**Affiliations:** 1 Department of Chemistry, University of Crete, Voutes Campus, GR-71003 Heraklion, Crete Greece; 2 Theoretical and Physical Chemistry Institute, National Hellenic Research Foundation, Vassileos Constantinou 48, GR-11635 Athens, Greece; 3 Department of Materials Science and Engineering, University of Crete, Voutes Campus, GR-71003 Heraklion, Crete Greece; 4 Materials & Manufacturing Directorate, Air Force Research Laboratory, Dayton, Ohio 45433 United States; 5 Universal Technology Corporation, Dayton, OH 45432 United States

**Keywords:** Electronic properties and devices, Electronic structure

## Abstract

The structural and electronic properties of molecularly pillared graphene sheets were explored by performing Density Functional based Tight Binding calculations. Several different architectures were generated by varying the density of the pillars, the chemical composition of the organic molecule acting as a pillar and the pillar distribution. Our results show that by changing the pillars density and distribution we can tune the band gap transforming graphene from metallic to semiconducting in a continuous way. In addition, the chemical composition of the pillars affects the band gap in a lesser extent by introducing additional states in the valence or the conduction band and can act as a *fine* band gap tuning. These unique electronic properties controlled *by design*, makes Mollecular Pillared Graphene an excellent material for flexible electronics.

## Introduction

During the last seven decades there has been a huge growth in the development of new electronic devices, which enabled for the development of new technologies and new markets globally. The discovery of new electronic materials and new methodologies to manipulate them has played a key role for the construction of the electronic parts needed for a chip. Among other materials included in a chip, semiconductors have attracted a lot of attention from the scientific community and the industry and significant efforts have been made to find new semiconducting materials to replace silicon. The basic requirements for the evolution of the silicon based integrated circuits have been the continuous increase in the speed, the minimization of the size and energy consumption and the reduction of the cost. Besides the efforts to improve the performance with respect to the above mentioned factors, there have been a lot of efforts to build electronic devices with better mechanical flexibility, giving rise to an emerging research field of flexible and stretchable electronics^1^. Such materials, which can be manufactured in various forms and shapes are expected to contribute significantly to develop modern electronic devices which are used in a variety of applications, such as telecommunications, sensors, imaging, energy conversion and batteries.

Flexible electronic materials is considered as one of the most emergent research areas nowadays since there is a tremendous need to build low cost, large area electronic devices with the ability to be incorporated on flexible substrates. They can be categorized as either inorganic or organic based materials with their own unique properties in terms of intrinsic flexibility, carrier density and carrier mobility. In order to build a flexible device, they must be deposited on flexible substrate, which usually is a polymer like PDMS, using a variety of techniques^2^. Reduction of dimensions and designing new flexible architectures give the ability to maintain the desired electronic properties and enhance the mechanical flexibility in the case of inorganic based flexible devices^3^. Organic based materials hold much of the promises to build flexible electronic devices since they have larger intrinsic flexibility with respect to inorganic based materials and it is easier to integrate them on flexible substrates^4,5^. Although significant progress has been made in controlling their electronic properties, their broad applicability suffers from their low chemical stability which increases the overall costs since special care should be considered during integration.

The great challenge in the area of flexible electronics is to overpass the barrier that the combination of the two different faces in the material put, i.e., desirable electronic properties as well as mechanical conformability. From device design perspective, the real novelty will be to design a relatively flexible material with pronounced electronic properties and not to combine two materials with completely different nature -a thin film transistor and a flexible plastic substrate. Such a design will lead to minimizing contact issues (heat dissipation, contact resistance) associated with the interface.

Among potential flexible alternatives to conventional electronics, different nanocarbon polymorphs, such as graphene, carbon nanotubes, carbon nanofibers, and nanographite have been a subject of special interest for the last decade. In particular, graphene, the ultimate two-dimensional material possesses fascinating electronic, mechanical, and thermal properties^6^. Graphene has a very large surface area (2630 m^2^g^−1^ from theoretical calculations)^7^, has large carrier mobility up to 200,000 cm^2^V^−1^s^−1^ when the carrier density is ~10^12^ cm^−2^ ^8,9^, possess highest value of room temperature electrical conductivity in room temperature (106 S cm^−1^)^10^, possess extreme mechanical properties such as the Young modulus (~1 TPa)^11^ and tensile strength (~42 Nm^−1^)^11^, and is known to be one of the best thermal conductors (thermal conductivity 3000–5000 W m^−1^ K^−1^)^12–16^ along in-plane direction. Its possible applications have triggered numerous studies over the past decade on how these properties are modified when doping and functionalization take place to the nanoscale.

One key feature which has limited graphene usage in nanoelectronics is the observation that it is a zero band gap material with the characteristic Dirac cones observed in the band structure. Graphene applications in electronics can be further expanded if graphene be efficiently converted to a semiconductor without great loss of aforementioned properties. It would be desirable to be able to tune its electronic properties in a controllable way by either tailoring its structure in the atomistic level or by applying external stimuli (mechanical or electrical fields). Structure modifications in graphene has been applied in sever always including the introduction of vacancy defects with varying sizes, the substitution of carbon atoms with heteroatoms, the construction of graphene nanoribbons with certain size, shape and chemical functionality on the edges, and the chemical functionalization of the basal plane of the graphene flakes. It has been shown that such modifications can effectively adjust the electronic properties of the corresponding materials.

Based on graphene structure and the large number of existing routes towards its chemical functionalization, novel 3D graphene architectures can be designed by incorporating various types of nano-constituents in between graphene layers. The incorporated spacers can keep the layers apart either by forming van der Waals heterostructures or by forming heterostructures through the formation of chemical bonds between the spacer and the 2D layers^17,18^. Pillared graphene, recently proposed by our group^19^, is such a nano-architecture, where carbon nanotubes are incorporated between graphene flakes to form a 3D carbon based porous nanostructure. Such nanostructures possess notable hydrogen storage, gas separation, electronic, thermal, and mechanical properties^20–24^ which could be tuned by tailoring the structure. Such interconnected graphene is still expected to be semi-metallic due to overwhelmingly large sp^2^ nature of carbon atoms, providing extended electronic cloud.

**Figure 1:**
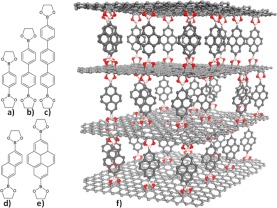
2D representation of the molecules considered as pillars for the design of MPG structures: benzene (**a**), biphenyl (**b**), triphenyl (**c**), naphthalene (**d**), pyrene (**e**). (**f**) 3D representation of an MPG architecture with graphene layers pillared by pyrene through the formation of 5-membered boroxine rings. C, O and B atoms are represented as grey, red and pink spheres respectively. H atoms have been omitted for clarity.

On the contrary, interconnecting graphene via organic linkers (we call it *molecular* pillaring) disrupt electron resonance in out-of-plane direction and can be used as a knob to tune the electronic behavior of graphene (semi-metallic to semiconducting) while providing the unique structural properties of a flexible material with the endless variety of *pillaring* organic molecules. In addition to tuning electronic behavior, molecular pillared graphene (MPG) (Fig. 1f) can be considered as a *platform* for building novel materials with highly tunable structural, thermal, mechanical, and surface properties that can be promising candidates for several applications including, electronics, optoelectronics, energy storage, catalysis, gas storage, and separation. Attempts toward this direction have already been reported in the literature concerning mostly materials focusing on gas adsorption and separation^25–34^. In some studies, synthetic routes toward molecular pillared architectures have been mentioned^14–16,35–37^ taking various forms of oxidized graphene as starting material. The graphene layers were interlinked though reactions of their oxygen containing groups with molecular precursors containing diboronic acid^14^, diamine^38^, di-isocyanate^26^ or bis-diazonium salt^39^ functionalities.

A similar strategy in materials science was successfully applied in Metal-Organic Frameworks (MOFs)^40^. In the *virtual space* of the thousands of MOF structures, whole families have been created sharing the same topology and altering the organic molecule acting as building bridge (linker). Similarly, in the graphene case, the number of organic molecules that can be used for pillaring graphene layers is virtually almost endless! Combining this with the variety of intramolecular lengths of the organic molecules and grafting patterns in graphene end up in a large family of novel 3-D architectures entitled as Molecular Pillared Graphene (MPG).

In order to explore the properties of MPGs in the present study, we designed fifteen novel flexible 3D graphene network structures. They were assembled by combining three different pillar densities with five highly aromatic molecules of different size and aromaticity. The structural and electronic properties of these 15 MPGs were examined by performing electronic structure calculations under the Tight Binding Density Functional Theory (TB-DFT) approximation. For electronic structure calculations, such an approximation at semi-classical level is necessary as full *ab-initio* calculations with such large number of atoms are extremely computationally demanding. Within DFTB framework, band gaps for all structures were calculated and the corresponding density of states (DOS) diagrams were analyzed to provide further insights into the origin of the states at the edges of the valence and conduction bands around the band gap. Such an analysis allowed us to systematically and quantitatively verify the effect of the pillared molecules and pattern on the electronic properties of each MPG structure. Moreover we examined the effect of the relative distribution of the molecular pillars on the band gap of these materials. Details for DFTB method and the corresponding calculations can be found in Methods section.

## Results

**Figure 2:**
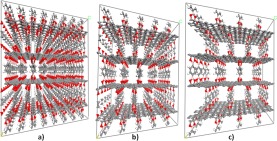
Supercells of the optimized periodic structures of MPGs with benzene acting as a pillar for the 3 pillar densities examined: (**a**) 1/8 (highest density), (**b**) 1/18, and (**c**) 1/32 (lowest density).

The design of the MPG structures was based on the variation of two factors. The first was the amount of the pillaring molecules with respect to the total number of carbon atoms of the graphene layers (density of pillars) and the second was the choice of the organic molecule which acted as the pillar. Three pillar densities were taken into account in the construction of the molecular models denoted as 1/8, 1/18 and 1/32. 1/8 corresponds to the densest packing of pillars considered in this study with one pillar per eight carbon atoms belonging to the graphene layers of the model. The difference in the pillar density can be easily seen in periodic models presented in Fig. 2. The selected pillar densities were combined with five aromatic organic molecules considered as pillars: phenyl, naphthalene, biphenyl, pyrene and triphenyl (Fig. 1a–e). Each of them was oriented with its long axis vertical to the plane of the graphene layers. The pillars were covalently bonded to the graphene through the formation of five-member boroxine rings as originally synthesized in the Graphene Oxide Framework (GOF)^14^ and Covalent-Organic Framework (COF) materials^41^. The rings are formed from the condensation reaction of the boronic acids of the organic pillars with the oxygen species on the basal plane of the oxidized graphene and include the bonding of a neighboring pair of carbon atoms of the graphene with the corresponding oxygen atoms of the boronic acid. The molecular supercells are consisted of two graphene layers with adjusted size depending on the pillar density and the length of the pillaring molecule. Initially, the pillars were orderly dispersed over the two interlayer spaces that are formed. Periodic boundary conditions were applied to transform the molecular model to the corresponding periodic structure (Fig. 2a–c).

The initial MPG periodic structures were constructed based on a hexagonal lattice (a = b, c, α = β = 90, γ = 120). In most cases, the features of the hexagonal lattice were retained with negligible distortions after structure and lattice optimization. The largest distortions (approximately ± 6°) were found for the case of the naphthalene acting as the pillar between graphene layers, independent of the density of the pillars in the structure. These distortions were attributed to the distortions occurred on the boronic ring due to the slight non-linearity of the naphthalene pillar. In Fig. 2, supercell geometry of the optimized periodic structures corresponding to the benzene derivatives for the three pillar densities can be seen. All geometries are available by the authors upon request.

The flexibility of the MPG’s was estimated by calculating the bulk and the shear modulus. We found that MPG’s flexibility is much larger than other materials such as bulk silicon or graphene. It becomes larger by increasing the length of the pillar or by decreasing the amount of pillars per C atoms of the graphene layers in the cell (see Supplementary Information).

**Figure 3:**
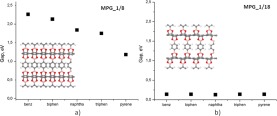
Band gap variation as a function of the chemical structure of the pillar for (**a**) 1/8 and (**b**) 1/18 density of pillars.

The effect of structural variation on the electronic properties of the initially designed MPGs was examined by performing calculations with the SCC-DFTB methodology as detailed in Methods section. Figure 3 present two plots of the band gap variation as a function of the pillar density (MPG_1/8 and MPG_1/18 in Fig. 3a,b respectively) and the chemical composition of the pillaring molecule. It can be clearly demonstrated that the band gap of MPGs can be tuned by varying both the pillar density and the chemical composition of the pillars. Introducing covalently bonded organic pillars lead to disappearance of Dirac cone and introduce a certain gap between conduction and valence bands. The density of the pillars was proved to be the dominating structural property for the determination of the band gap and the modulation of the electronic properties. Their properties can be altered from semiconducting to almost conducting.

For the three pillar densities that were considered here for the MPG structures designed initially, we found that the band gap decreases by decreasing the pillar density (less pillars per graphene surface area). The reduction in the band gap happens with a fluctuation for the intermediate pillar density (1/18) which is caused by the non isotropic relative distribution of the pillars belonging to the adjacent interlayer spaces which can be considered in the periodic structures. The semiconducting behavior can be attributed to the disruption of the extended π-system of graphene layer in all directions due to the presence of sp^3^ hybridized carbon atoms resulting from the formation of the boronic rings. Fewer pillars (low pillar density) lead to fewer sp^3^ hybridized carbon atoms and a smaller disruption of the semi-metallic properties of the graphene, where larger number of pillars will partially destroy the graphene electronic properties and lead to semiconducting behavior. For the lowest pillar density (MPG_1/32), the band gap for the corresponding structures was found to be 0.98 eV with a small variation for the case of the pyrene derivative (MPG_1/32_pyrene) for which the value of 0.71 eV was calculated. For the intermediate pillar density of 1/18, the band gap was found to be at 0.14 eV.

The effect of the chemical composition of the pillars on the band gap values was found to be less profound than the pillar density, especially for the cases corresponding to the 1/18 and 1/32 pillar densities. The difference can be easily observed when comparing the band gap values as a function of the chemical composition for 1/8 and 1/18 pillar densities in Fig. 3a,b. The band gap for the 1/8 density varies from 1.18 eV to 2.26 eV for the cases of pyrene and benzene derivatives respectively. In this case the band gap can be modulated by ~1 eV by altering the nature of pillaring molecules. In the cases of 1/18 and 1/32 densities, the pillar composition does not affect the band gap as described earlier when discussing the effect of pillar density.

**Figure 4:**
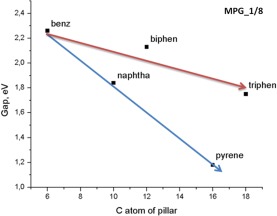
Band gap variation of MPG as a function of the number of C atoms of the pillar molecule for 1/8 pillar density. Arrows show the trends of the groups.

For the most interesting 1/8 pillar density, we plotted the band gap with respect of the C-atoms per pillaring molecule (Fig. 4). Two different trends were identified regarding to the pillar composition from isolated (phenyl, biphenyl, and triphenyl, red line) or fused benzene rings (naphthalene and pyrene, blue line). As seen from the figure, a steepest slope of the band gap was observed in the case of fused rings which cannot be attributed to the continuous increase of parallel p orbitals as the number of adjacent carbon atoms increase, because similar trends should be existing for the other two pillar densities. We argue that the origin of this phenomenon comes from the overlapping of π systems belonging to adjacent pillars and the different trend between isolated and fused rings arises from the relative size of the molecules and the corresponding degree of the overlapping.

The influence of the atoms belonging either to the pillar or the graphene layer on the electronic properties of the MPG structures was further explored by plotting the total density of states (DOS) and the partial density of states (PDOS) for all structures in this study. Here, we are mainly interested in locating the contributions of the carbon, boron, and oxygen atoms of the structures at the states near the bottom of the conduction band and the top of the valence band. Hydrogen has been excluded from the plots since it is expected to have no contributions on the area of DOS plots that we are interested in. Also the s type atomic states of the carbon, boron and oxygen have no contribution to the valence or the conduction band, as expected, and thus are not considered here.

**Figure 5:**
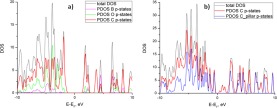
(**a**)Density of States (DOS) per unit energy for the p states of all the atoms of the MPG_1/8_benz. (**b**) DOS plot of the p states of all C atoms and the C atoms of the pillar for the MPG_1/18_pyrene. The Fermi energy has been shifted to zero.

From the examination of the DOS plots for the initial MPG structures, we arrived at two general conclusions: (a) The main contributions in the states around the Fermi energy come from the p-type states of the carbon atoms, with smaller or negligible contributions from p-type states of the oxygen or the boron atoms (Fig. 5a,b) the origin of the p-states at the edges of the valence (VB) and the conduction (CB) bands depend both from density of the pillars and the chemical composition of the pillars. In the case of 1/8 pillar density, the p-states at the edge of VB belong to C atoms of the pillar where the corresponding states in the CB to C atoms of the graphene. For the pyrene derivative, small contributions from the C atoms of the pillar are also present in CB. The introduction of more p states in the VB as a function of the number of C atoms in conjunction with a shifting of their energies closer to the Fermi energy due to the proximity of the pillars further supports our previous observation about the gradual decrease of the band gap. For 1/18 density, the p-states in the edges of the VB and CB are contributed from C atoms of the graphene, except from the pyrene derivative where some of the p states in the VB are also introduced from the C atoms of the pillars (Fig. 5b). This explains the ineffective modulation of the band gap by altering the variation of chemical composition of the pillar. The DOS plots for MPG_1/32 derivatives shows that p states of the VB and CB are dominated from C atoms of graphene for the benzene derivative where forthe other derivatives, the states at the edge of the VB are contributed almost equally from C atoms of the pillar and graphene.

In order to further investigate the origin of the band gap variation we performed analogous calculations to single graphene layers functionalized similarly with the pillared structures. We used phenyl and pyrene derivatives of the corresponding MPG structures to build the functionalized monolayers (Fig. S25 in the Supplementary Information). Our calculations showed that the band gap values are similar for the functionalized monolayers and the three dimensional MPG structures with the same pillar density and the same pillar composition. It was confirmed that the effect on the band gap is due to the functionalization and not the linking. This is a very important observation for the MPG material fabrication process since it gives the freedom of constructing only a few layer device and combine the superior electronic properties with the desired mechanical stability.

We also examined the effect of the pillar distribution in MPG materials, by constructing periodic models with a non isotropic pillar arrangement. For this we considered the phenyl derivative of MPG materials for the 1/18 and 1/32 pillar densities. The construction of the periodic models was followed by the optimization of atomic positions and the lattice parameters of the cells and the calculation of the band gaps. Our calculations revealed that the distribution of the pillars can also affect the band gap and tune the electronic structure of MPG materials from semiconducting to metals. For the different pillar configurations that were examined (see Supplementary Information), we found band gap values ranging from 1.39 eV to gapless for the case of 1/18 pillar density, where for 1/32 density, the variation was found to be from 0.98 eV to gapless.

## Discussion

We have examined a set of molecularly pillared graphene networks by varying both the pillar density and the chemical composition of the pillar molecule in terms of electronic properties by performing TB-DFT calculations. Our results show that by changing the pillar density, we can tune the band gap transforming graphene from metallic to semiconducting in a controlled manner. For the highest pillar density, the chemical composition of the pillars also affects the band gap by introducing p-states in the valence band, where this happens to a lesser extent for the other two lower pillar densities. We also showed that the distribution (isotropic or not) of the pillars can induce a variation in the band gap, transforming MPG materials from semiconducting to conducting. The key advantages of these materials compared to a functionalized single graphene layer are the additional mechanical stability of MPG’s due to the linking of the graphene layers by pillars, the engineering of the graphene layer relative position and the corresponding interspace. We believe that this unique way of tuning electronic properties, controlled by design, makes the proposed organically linked graphene 3D nanostructures an excellent alternative for flexible electronics. Considering the thousands of organic linkers that can be used for pillaring graphene, we have presented this study as a ‘proof of principle’ and expect that this study will pave the way for a novel large family of flexible electronic materials.

## Methods

The atomic positions and the lattice vectors of all MPG periodic structures were preoptimized by performing molecular mechanics calculations. The UFF^42^ forcefield was used along with the conjugate gradient (CG) algorithm for the optimization in the P1 symmetry. These calculations were done with the GULP^43^ code. Then, periodic optimization (both atom positions and lattice vectors) were carried out by using Self-Consistent-Charge Density-Functional-Based Tight-Binding (SCC-DFTB)^44–46^ method. This method is based on a second order expansion of the Kohn-Sham total energy in DFT with respect to charge density fluctuations and is capable of maintaining all the qualities of DFT without extensive empirical parametrization. Periodic boundary conditions were used to represent the crystalline solid state of the MPG structures. The conjugate gradient method was used for the geometry optimization, where the atomic force tolerance was set to 3∙10^−4^ eV/Å. No symmetry constrains were imposed during optimization. The number of k-points needed for the calculations were determined by reaching convergence of the total energy as a function of the k-points according to the scheme proposed by Monkhorst and Pack^47^. A 4 × 4 × 4 grid proved to be sufficient for sampling the Brillouin zone for all the periodic structures that were studied here. The necessary integrals together with other atomic and diatomic parameters for building the DFTB Hamiltonian were taken from matsci-0-3 Slater-Koster files^48^, which were downloaded from DFTB.org. matsci-0-3^49,50^ contain atomic parameters for the B and O atoms which were originally developed in order to correctly describe the structural and electronic properties of similar 2D COF materials^37^.

The electronic properties of the designed pillared graphenes were explored by calculating the total density of states (total DOS), partial density of states (PDOS), and the band structure diagrams for the optimized periodic structures. For the k-point sampling, a 4 × 4 × 4 Monkhorst-Pack scheme was used and the Self-Consistent-Charge tolerance was set to 1∙10^−5^ eV. In the DOS diagrams, the Fermi level on the horizontal axis was shifted to zero. Structure optimization and calculations of the electronic properties were performed with DFTB+ v1.2 and DFTB+ v17.1 codes respectively^51^.

## Supplementary information

suporting Information

## Data Availability

All relevant data are available from authors on request.
