# Supplementary material for: Organically interconnected graphene flakes: A flexible 3-D material with tunable electronic bandgap
Source: Sci Rep. 2019 Sep 23;9:13676. doi: 10.1038/s41598-019-50037-y (PMC6757027; doi:10.1038/s41598-019-50037-y)
Supplement: Supplementary file 1 — suporting Information [file 41598_2019_50037_MOESM1_ESM.docx]

**Supplementary Information for:**

**Organically interconnected graphene flakes: A flexible 3-D material with tunable electronic bandgap.**

Emmanuel Klontzas^a,b^, Emmanuel Tylianakis^c^, Vikas Varshney^d,e^, Ajit K. Roy^e^, George E. Froudakis^a*^

^a^Department of Chemistry, University of Crete, Voutes Campus, GR-71003 Heraklion, Crete Hellas.

^b^Theoretical and Physical Chemistry Institute, National Hellenic Research Foundation, Vasileos Konstantinou 48, GR-11635, Athens, Hellas

^c^Department of Materials Science and Engineering, University of Crete, Voutes Campus, GR-71003 Heraklion, Crete Hellas.

^d^Materials & Manufacturing Directorate, Air Force Research Laboratory, Dayton, Ohio 45433, United States

^e^Universal Technology Corporation, Dayton, OH, 45432, United States

E-mail: [frudakis@uoc.gr](mailto:frudakis@uoc.gr)

**Contents**

1. Design principles of MPG structures.
2. Report of the structural characteristics of the optimized cells for all MPG structures.
3. PDOS analysis of the contribution of carbon atoms belonging to the pillar for MPG_1/8_benz and MPG_1/8_pyrene.
4. PDOS plots of the initial fifteen MPG structures.
5. Non isotropic pillar distribution in the adjacent interspaces of MPG materials.
6. Computational details for the calculations on functionalized graphene monolayers.
7. Calculations on the flexibility of the initial fifteen MPG structures.
8. **Design principles of the MPG periodic structures.**

Unit cell of MPG’s contains two graphene layers and two pillars. One of them is placed in each interlayer space of the structures. The unit cells for all pillar densities were created by multiplying the initial four-carbon atoms unit cell by two, three and four times in the a and b axes of the unit cell. The increment of the size of the graphene layers (increment of the number of carbon atoms of the graphene layers in the unit cell) while keeping the same number of pillars in each unit cell led to structures with different pillar densities.

Afterwards the z components of the coordinates of carbon atoms of the graphene layers in the unit cell were adjusted to create the interlayer distance that fits best to the length of the pillar. Then, the pillars were inserted between the layers and they were oriented perpendicular to the graphene layers. For all the models, the pillars were orderly distributed in each of the interlayer spaces, considering that they must have almost the same distance from their first neighboring pillars if the unit cell would be replicated in space. A 3x3x1 representation of the unit cells for the three pillar densities that explored has been created (Figures S1 to S3) to give a better insight of the pillar distribution in each interlayer space. Measured distances in black color correspond to the distances between boron atoms of the boroxine rings of neighboring pillars.


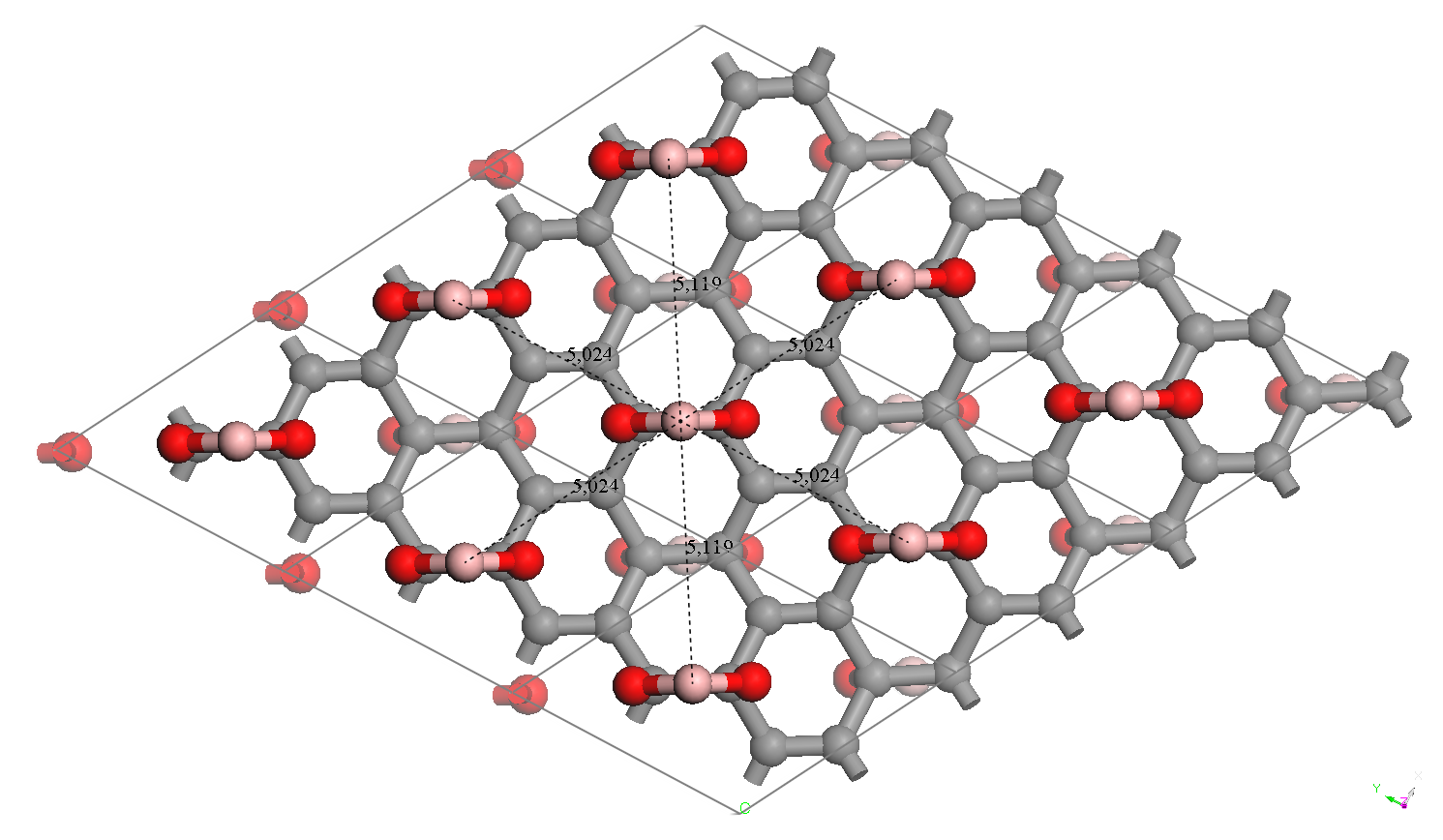


Figure S 1. 3x3x1 supercell of 1/8 MPG structure.


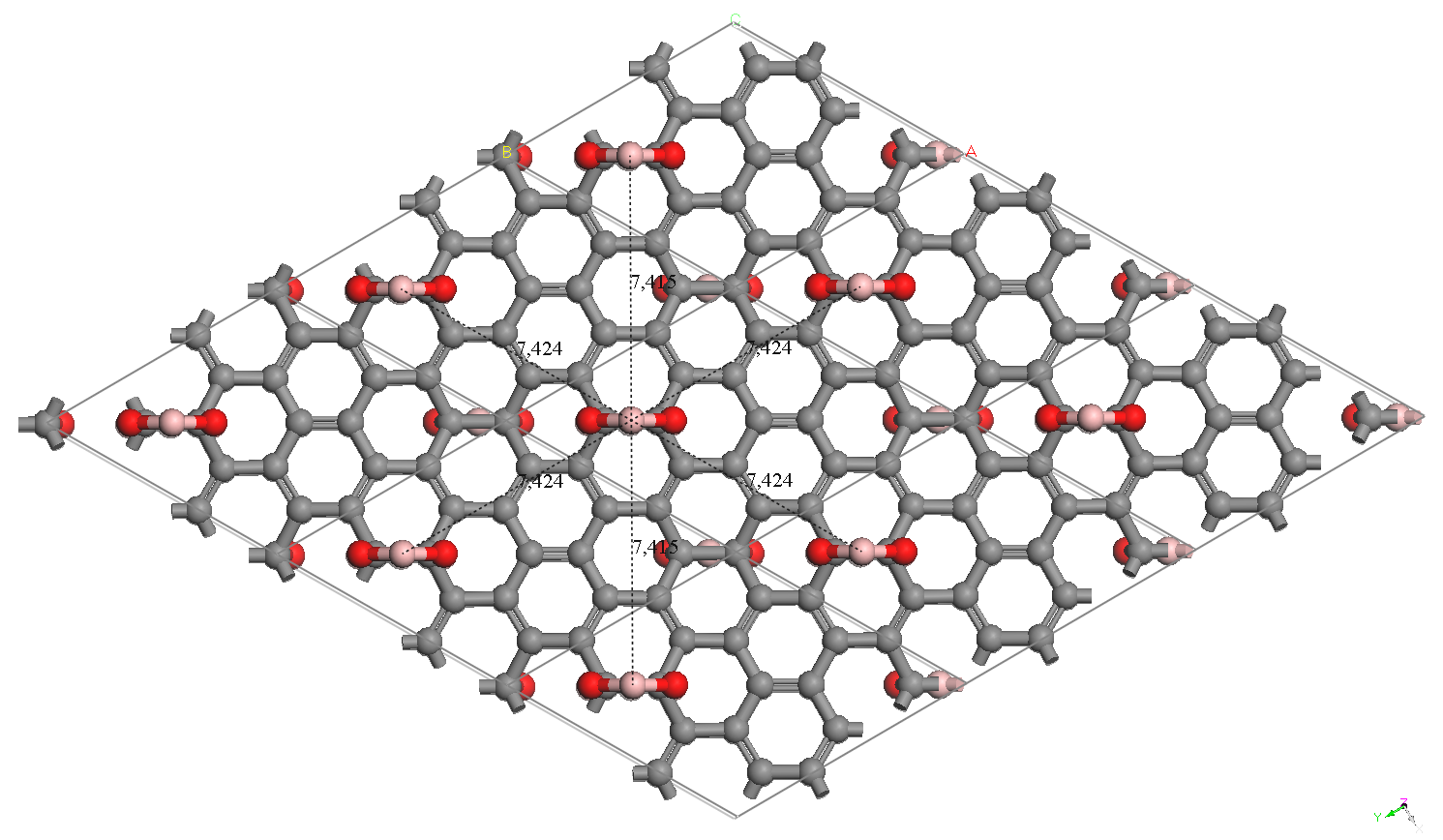


Figure S 2. 3x3x1 supercell of 1/18 MPG structure.


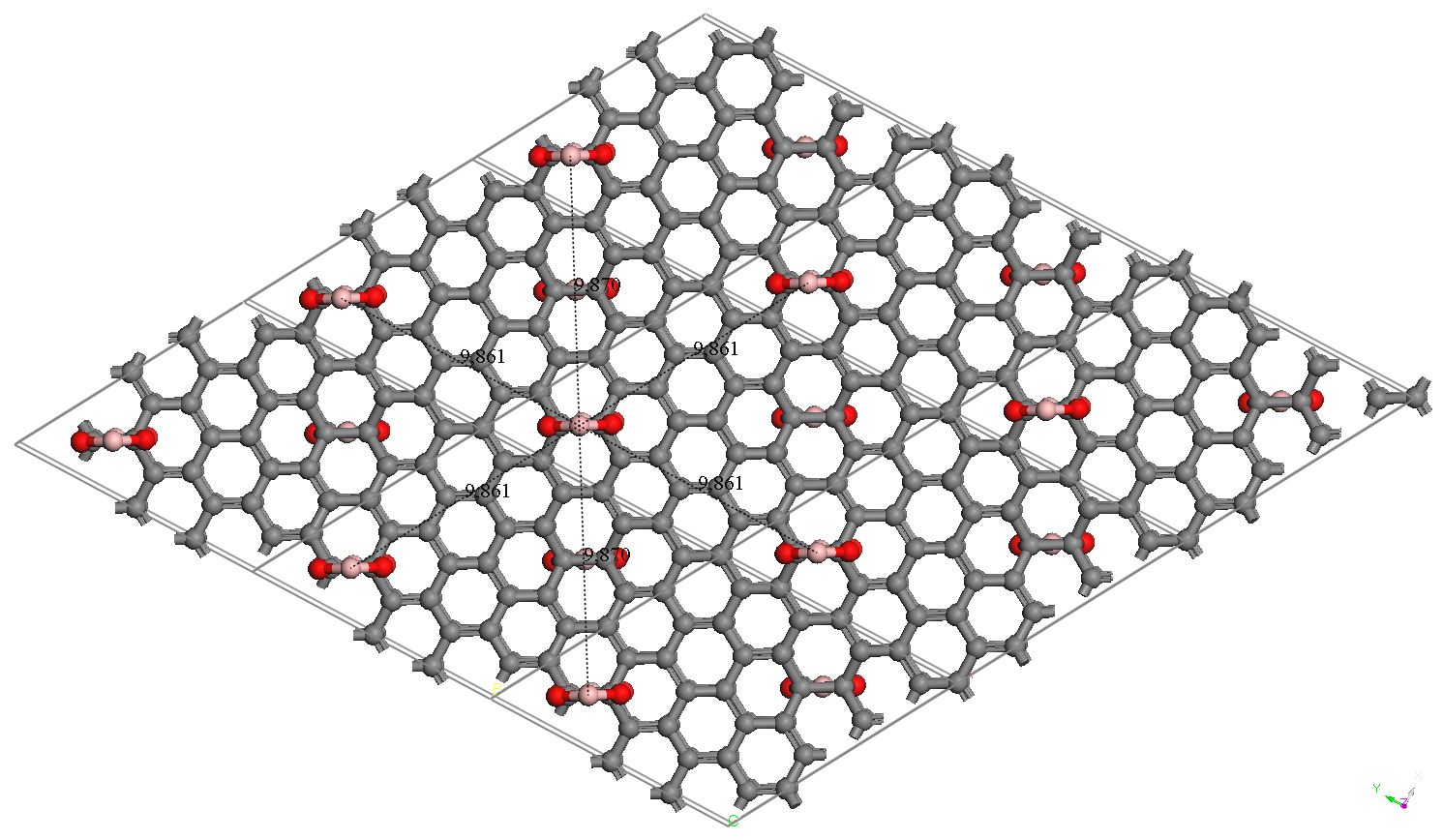


Figure S 3. 3x3x1 supercell of 1/32 MPG structure.

Another design parameter is the relative position of the pillars of the upper interlayer space with respect to those of the lower interlayer space. There is a distinction between 1/8 and 1/32 structures with respect to 1/18 pillar density concerning this parameter. This distinction can be better visualized if we consider the rhombus formed by four pillars of the same interlayer space and then locate the position of a pillar belonging to the lower interlayer space in the area of the rhombus. Details can be seen in the following figures representing 3x3x1 supercells of the structures (Figures S4 to S6). For 1/8 and 1/32 cases, the pillar is located in the center of the rhombus (denoted as cyan sphere), where in the case of 1/18 its position is shifted from that center (relative position of yellow sphere with respect to cyan sphere). Caron and hydrogen atoms of the pillars have been omitted for clarity.


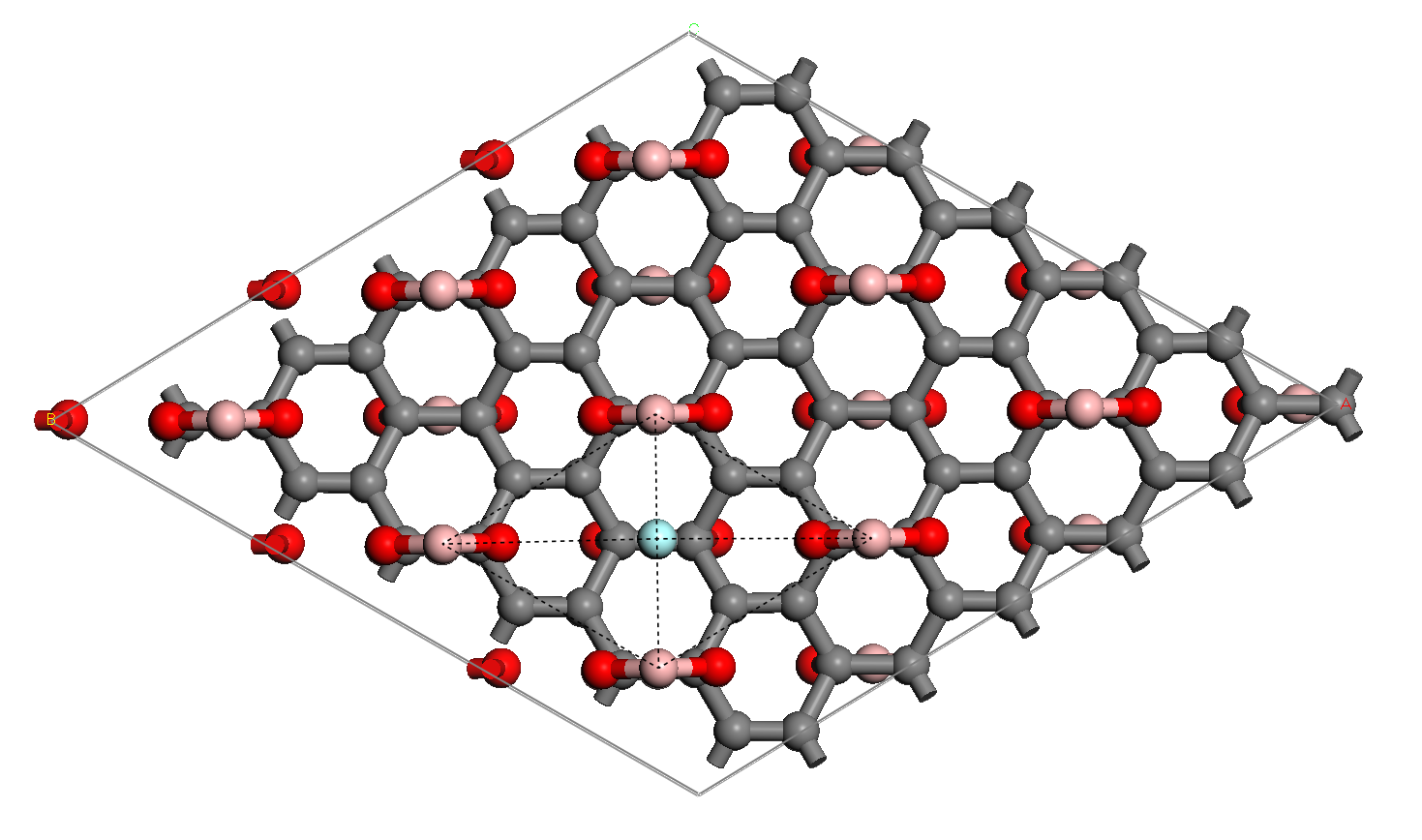


Figure S 4. 3x3x1 supercell of 1/8 MPG structure representing the relative position of the pillars belonging to different interlayer spaces.


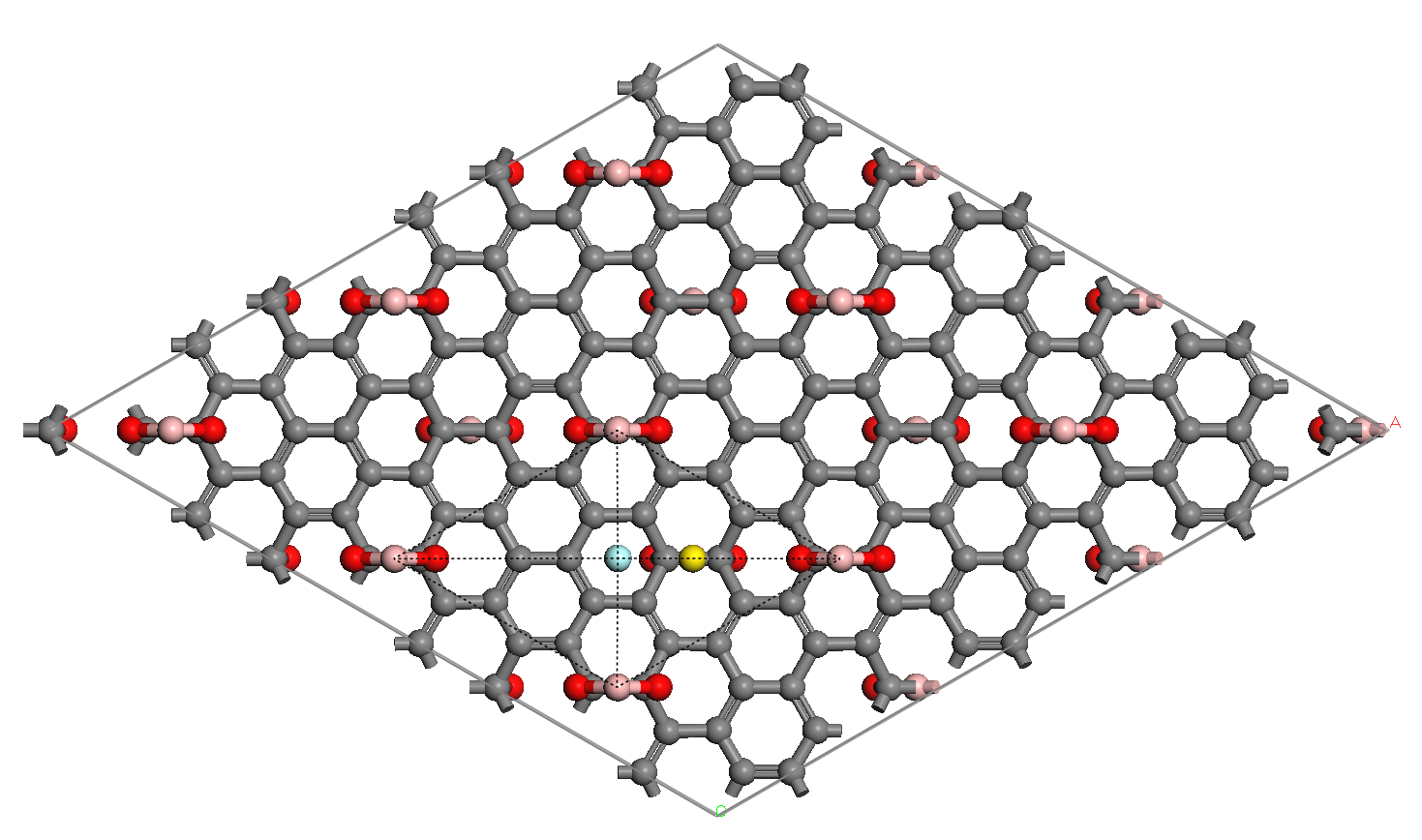


Figure S 5. 3x3x1 supercell of 1/18 MPG structure representing the relative position of the pillars belonging to different interlayer spaces.


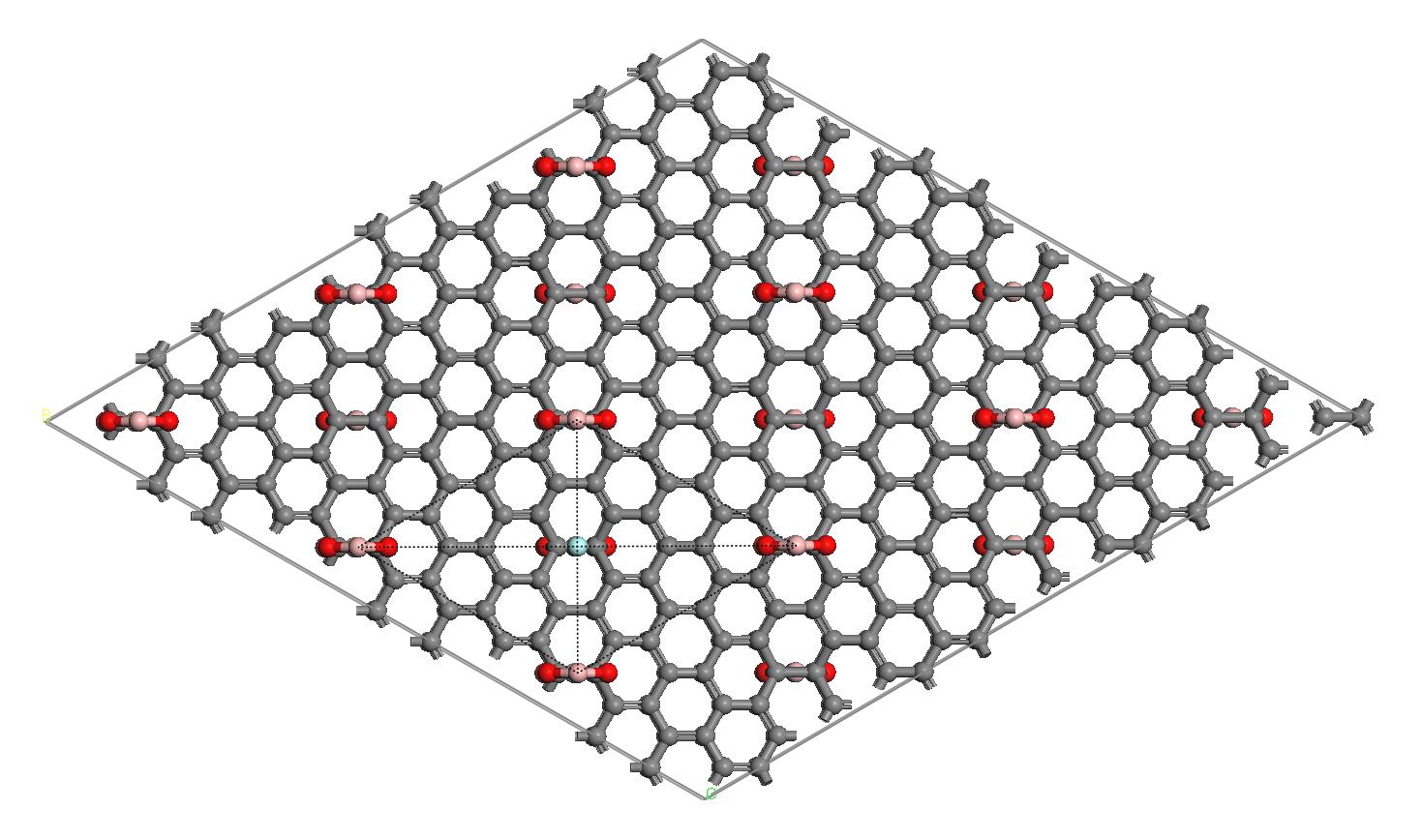


Figure S 6. 3x3x1 supercell of 1/32 MPG structure representing the relative position of the pillars belonging to different interlayer spaces.

1. **Report of the structural properties of optimized unit cells for all MPG structures in this study. Band gap has been also included.**

Table S1. Structural properties of the optimized unit cells for all the periodic structures that were studied including lattice parameters, number of C atoms that belong to the graphene layers in each unit cell, band gap and d spacing. The first column reports the characteristic name of the structure containing the corresponding density of the pillars. a, b, c and d spacing values are in Å, α, β, γ in degrees and band gap in eV.

|  | **a** | **b** | **c** | **α** | **β** | **γ** | **Number of C atoms belonging to graphene layers in a unit cell** | **Band Gap** | **d-spacing** |
| --- | --- | --- | --- | --- | --- | --- | --- | --- | --- |
| **MPG 1/8 benz** | **5.029** | **5.024** | **22.421** | **90.00** | **90.00** | **118.83** | **16** | **2.26** | **11.209** |
| **MPG 1/8 biphen** | **5.025** | **5.025** | **31.023** | **90.00** | **89.99** | **118.75** | **16** | **2.13** | **15.511** |
| **MPG 1/8 naphtha** | **5.029** | **5.029** | **26.803** | **86.09** | **93.89** | **118.89** | **16** | **1.84** | **13.360** |
| **MPG 1/8 pyrene** | **5.011** | **5.011** | **30.875** | **89.99** | **90.00** | **117.25** | **16** | **1.18** | **15.437** |
| **MPG 1/8 triphen** | **5.034** | **5.034** | **39.658** | **90.00** | **89.99** | **118.87** | **16** | **1.75** | **19.829** |
| **MPG 1/18 benz** | **7.424** | **7.424** | **22.959** | **89.99** | **90.00** | **120.08** | **36** | **0.14** | **11.479** |
| **MPG 1/18 biphen** | **7.424** | **7.424** | **31.569** | **89.99** | **90.00** | **120.08** | **36** | **0.14** | **15.784** |
| **MPG 1/18 naphtha** | **7.424** | **7.424** | **27.333** | **85.89** | **94.11** | **120.08** | **36** | **0.13** | **13.620** |
| **MPG 1/18 pyrene** | **7.424** | **7.424** | **31.460** | **89.99** | **90.00** | **120.08** | **36** | **0.14** | **15.730** |
| **MPG 1/18 triphen** | **7.424** | **7.424** | **40.178** | **90.00** | **89.99** | **120.08** | **36** | **0.14** | **20.089** |
| **MPG 1/32 benz** | **9.860** | **9.861** | **23.45** | **90.00** | **89.99** | **119.94** | **64** | **0.98** | **11.725** |
| **MPG 1/32 biphen** | **9.860** | **9.860** | **32.065** | **89.99** | **90.00** | **119.94** | **64** | **0.97** | **16.032** |
| **MPG 1/32 naphtha** | **9.860** | **9.860** | **27.834** | **85.84** | **94.17** | **119.94** | **64** | **0.98** | **13.868** |
| **MPG 1/32 pyrene** | **9.861** | **9.861** | **31.949** | **89.99** | **90.00** | **119.94** | **64** | **0.71** | **15.975** |
| **MPG 1/32 triphen** | **9.860** | **9.861** | **40.668** | **90.00** | **90.00** | **119.94** | **64** | **0.98** | **20.334** |

1. **PDOS analysis of the contribution of carbon atoms belonging to the pillar for MPG_1/8_benz and MPG_1/8_pyrene.**


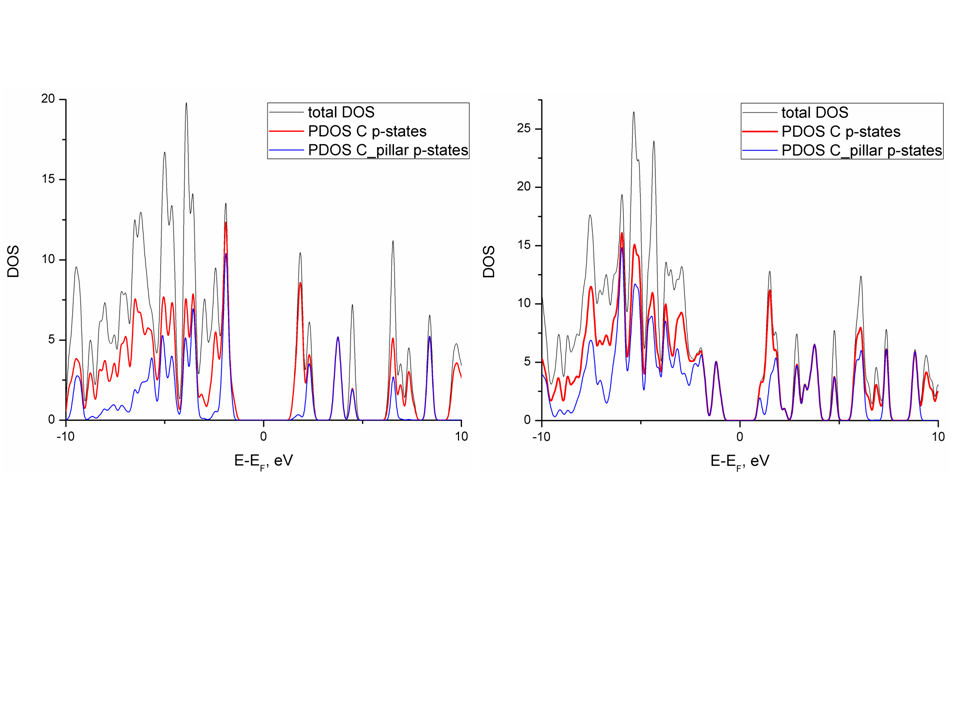


Figure S 7. PDOS analysis of the contribution of carbon atoms of the pillars for MPG_1/8_benz (left) and MPG_1/8_pyrene (right).

Figure S1 present the PDOS analysis of the carbon atoms of the MPG_1/8_benz and MPG_1/8_pyrene. Apart from the total DOS, PDOS of the carbon atoms of the unit cell and PDOS of the carbon atoms of the pillars are included for both cases. Only the p-states have been included in the plots since these shows the largest contribution in the area around the Fermi energy. For both materials, we observe that the states at the edge of the valence and the conduction bands manly arise from the carbon atoms of the structure, by comparing total DOS with the PDOS of carbon atoms of the structure (black and red lines respectively). Moreover, carbon atoms belonging to the pillars (blue line) has the largest contribution at the edge of the valence band where carbon atoms of the graphene layers has the largest contributions at the edge of the conduction bands for both benz and pyrene derivatives. In the case of pyrene derivative, carbon atoms of the pyrene pillars exclusively contribute the states of the valence band where they also have a larger contribution in the conduction band with respect to benzene derivative.

1. **PDOS plots of the fifteen MPG structures.**


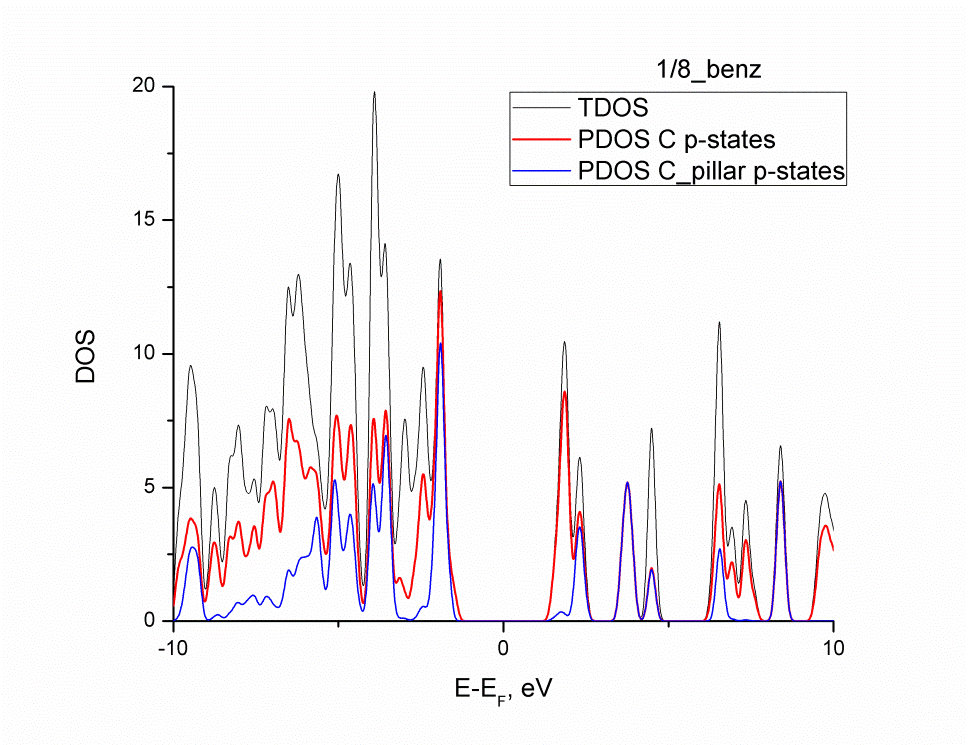


Figure S 8. PDOS plot for MPG_1/8_benz.


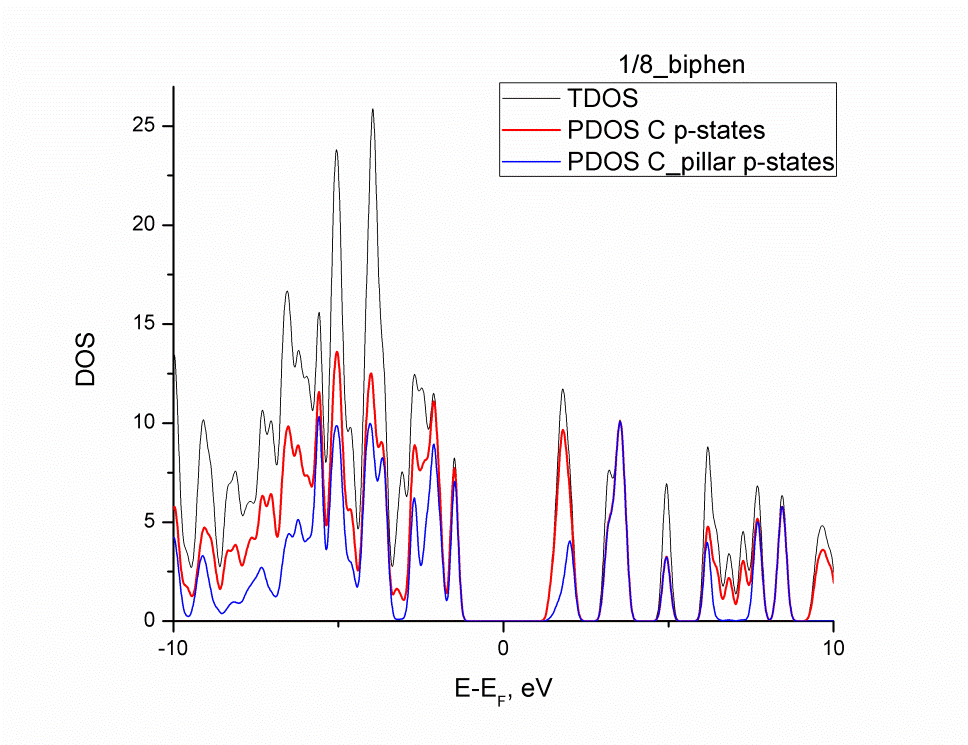


Figure S 9. PDOS plot for MPG_1/8_biphen.


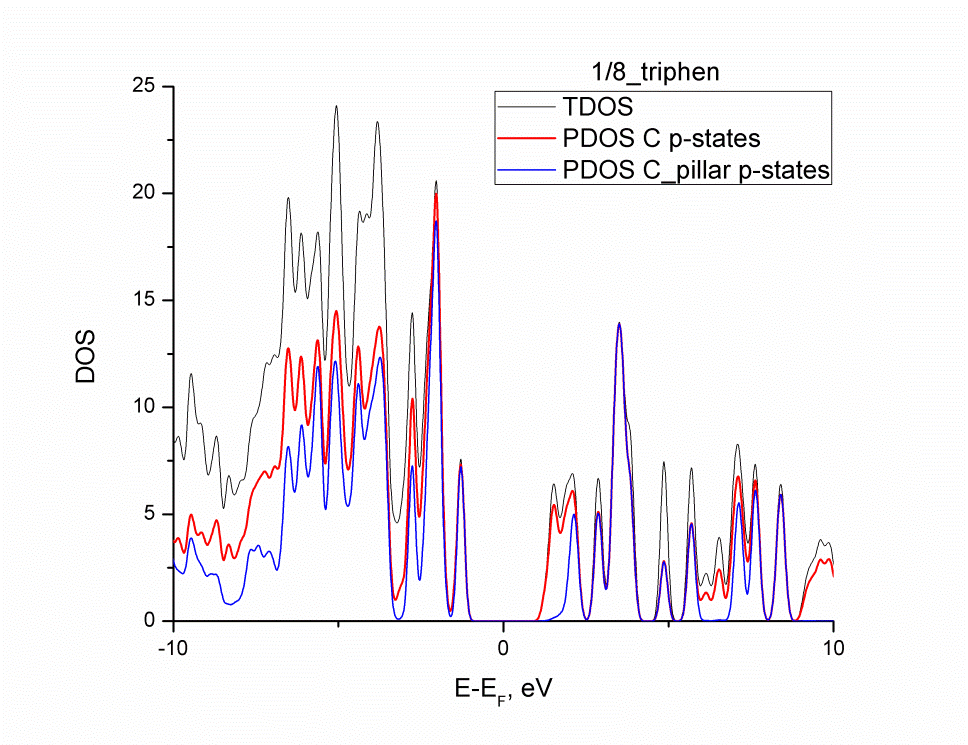


Figure S 10. PDOS plot for MPG_1/8_triphenyl.


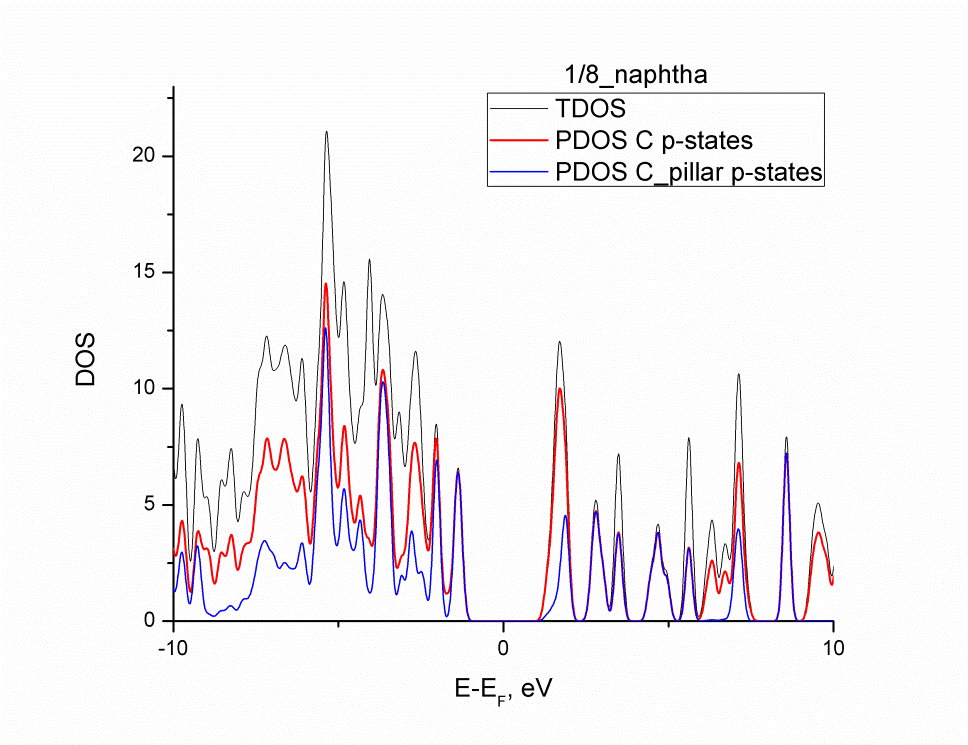


Figure S 11. PDOS plot for MPG_1/8_naphtha.


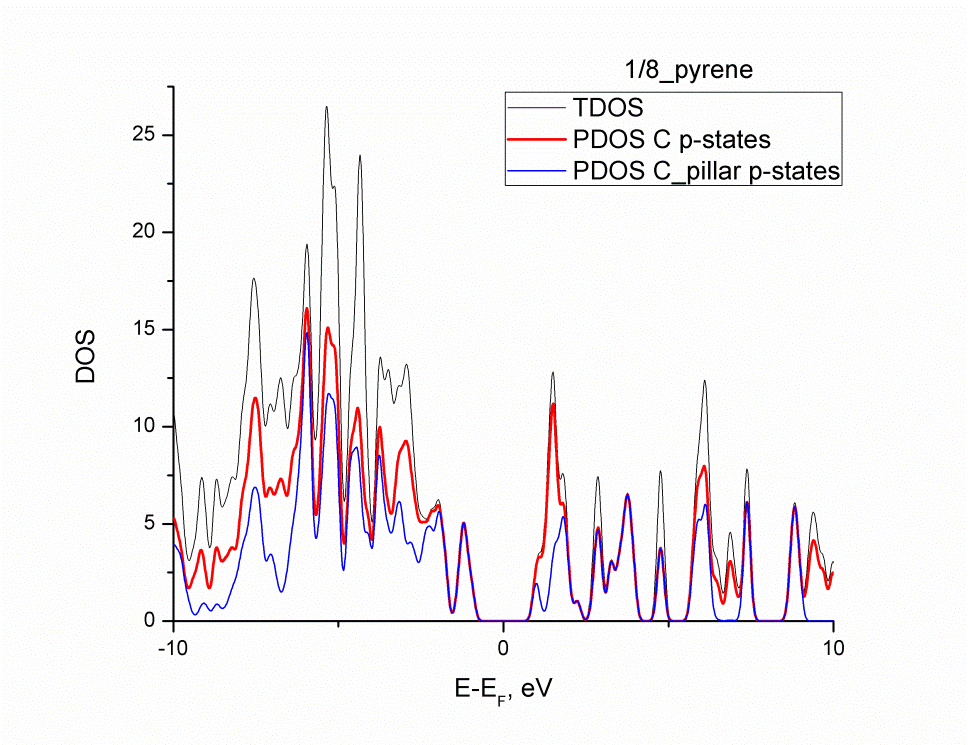


Figure S 12. PDOS plot for MPG_1/8_pyrene.


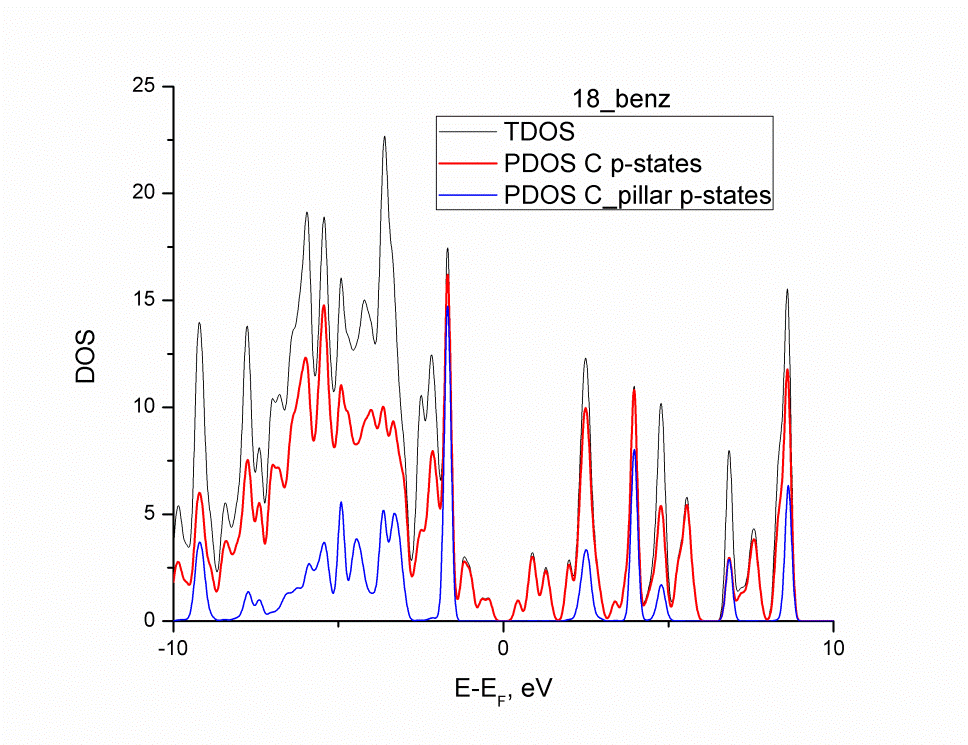


Figure S 13. PDOS plot for MPG_1/18_benz.


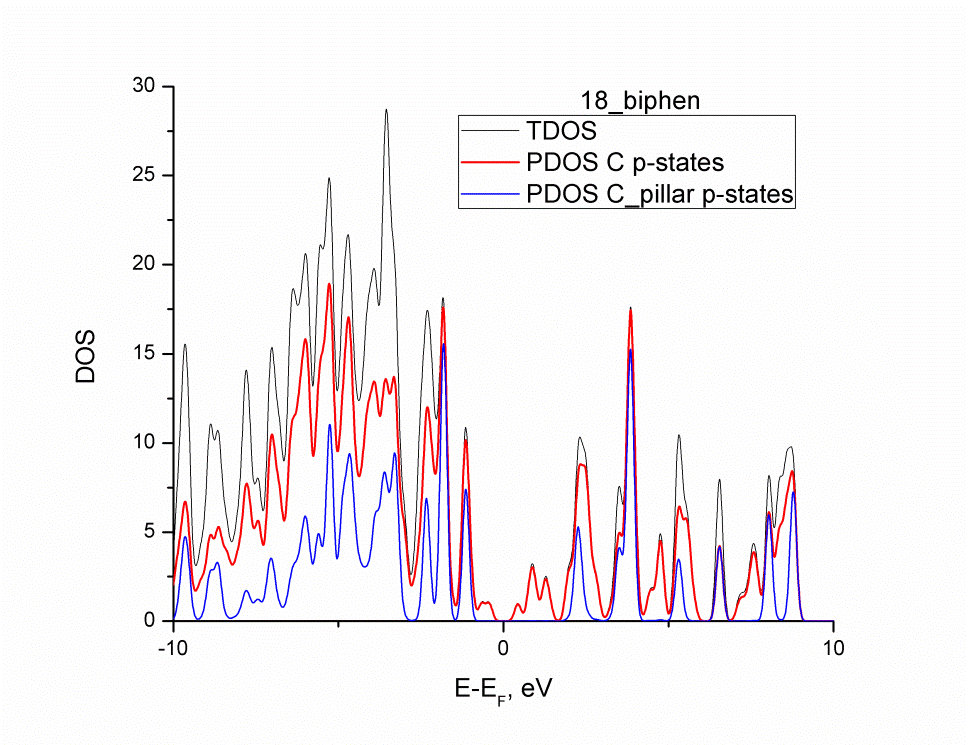


Figure S 14. PDOS plot for MPG_1/18_biphen.


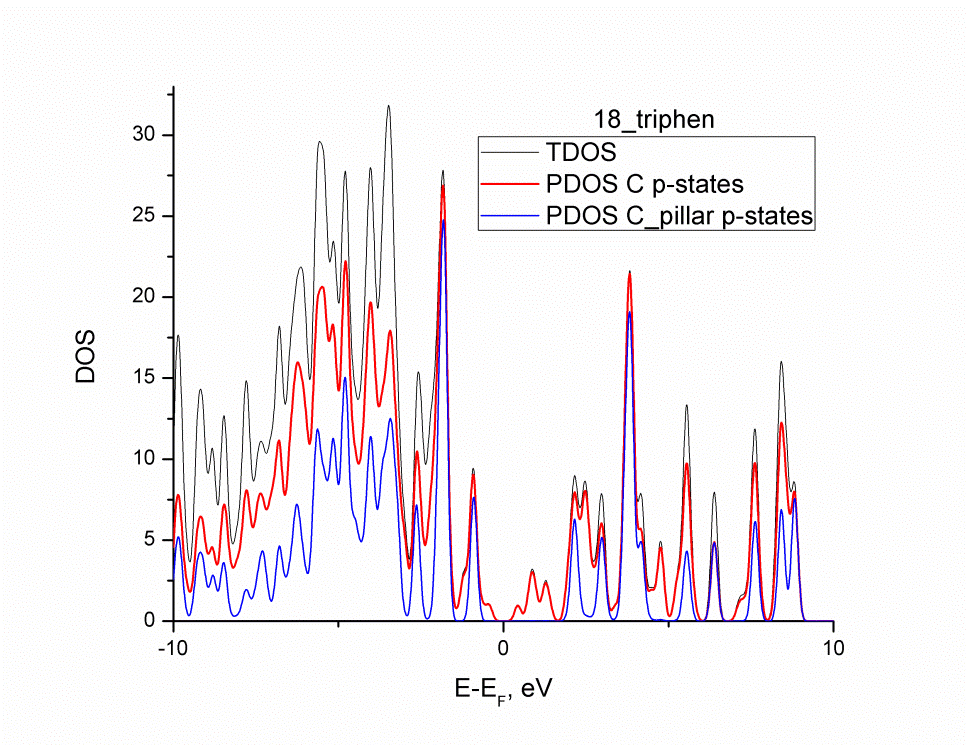


Figure S 15. PDOS plot for MPG_1/18_triphenyl.


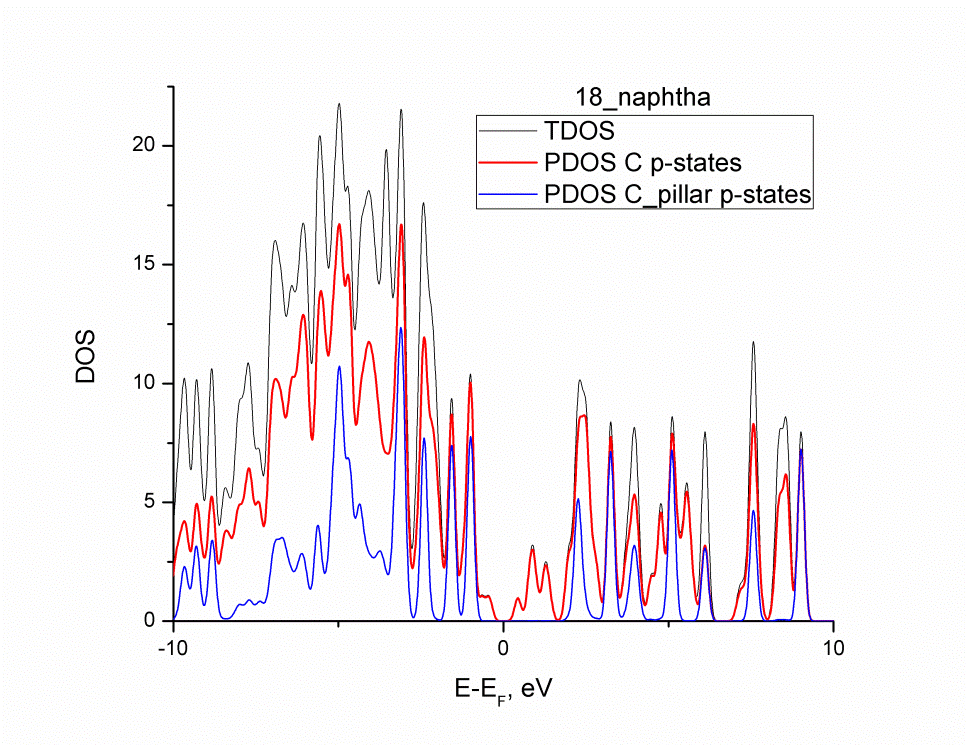


Figure S 16. PDOS plot for MPG_1/18_naphtha.


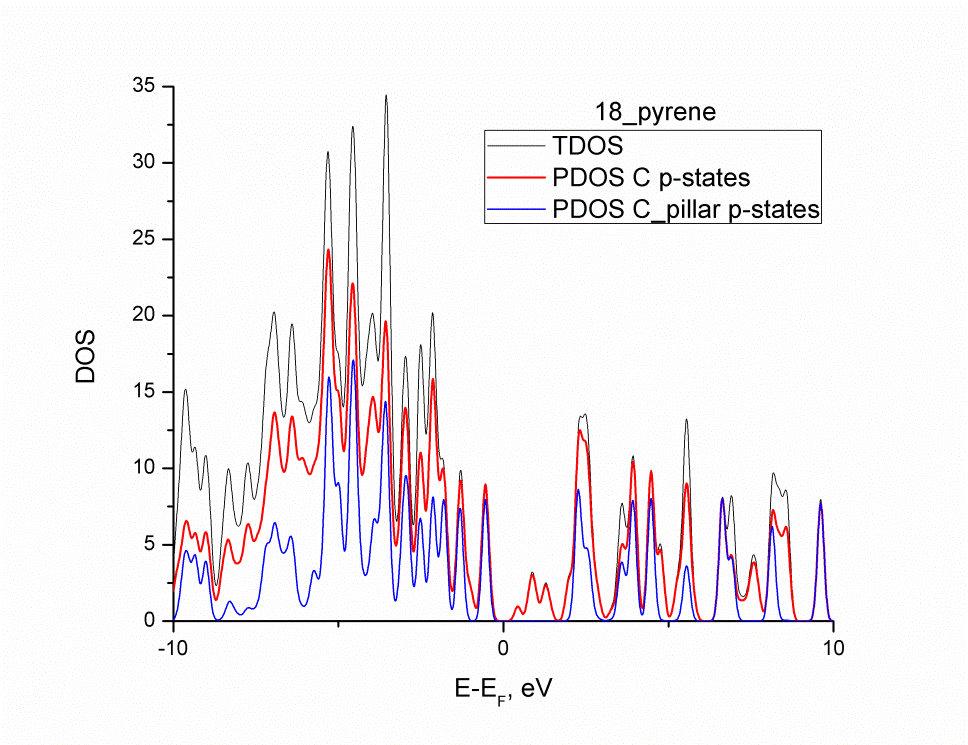


Figure S 17. PDOS plot for MPG_1/18_pyrene.


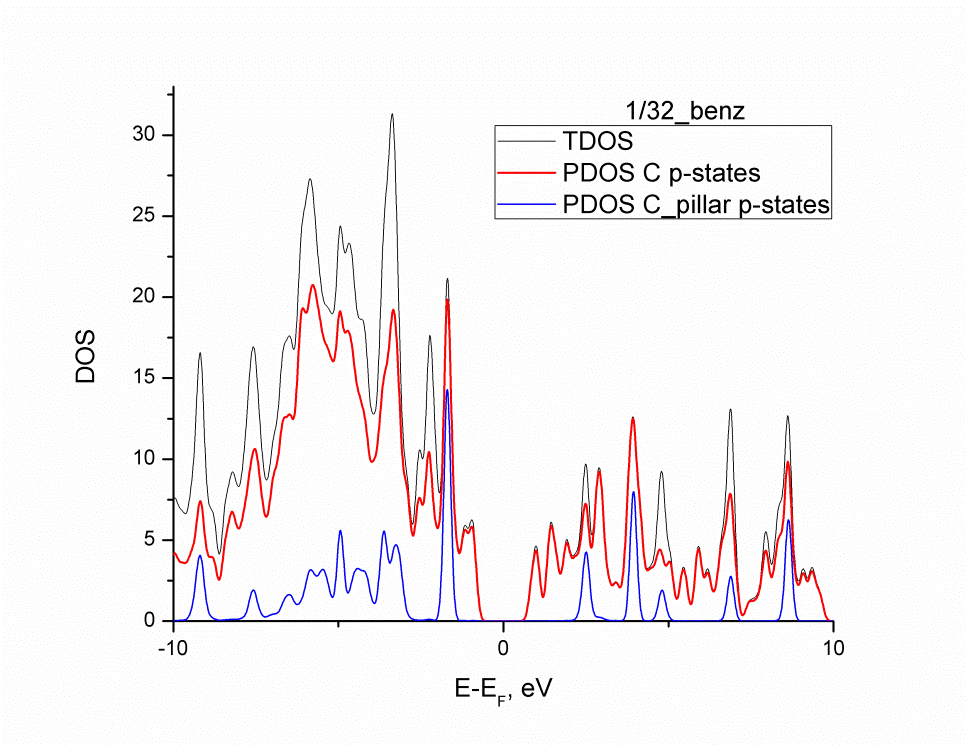


Figure S 18. PDOS plot for MPG_1/32_benz.


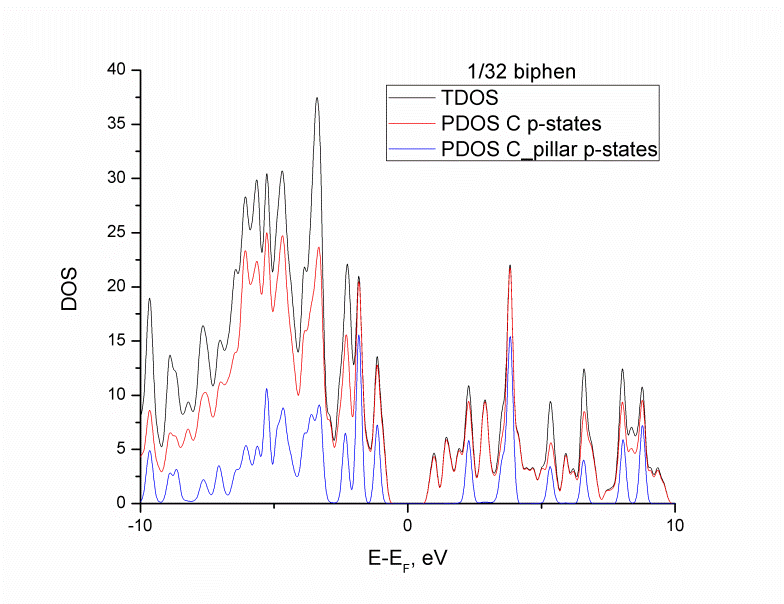


Figure S 19. PDOS plot for MPG_1/32 _biphenyl.


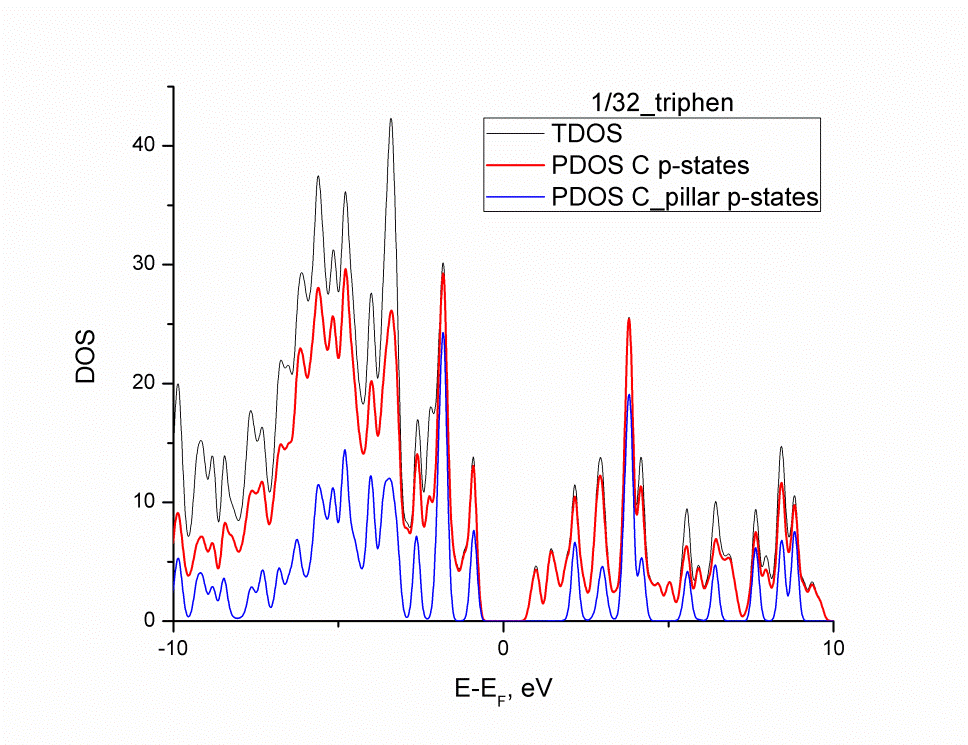


Figure S 20. PDOS plot for MPG_1/32_triphenyl.


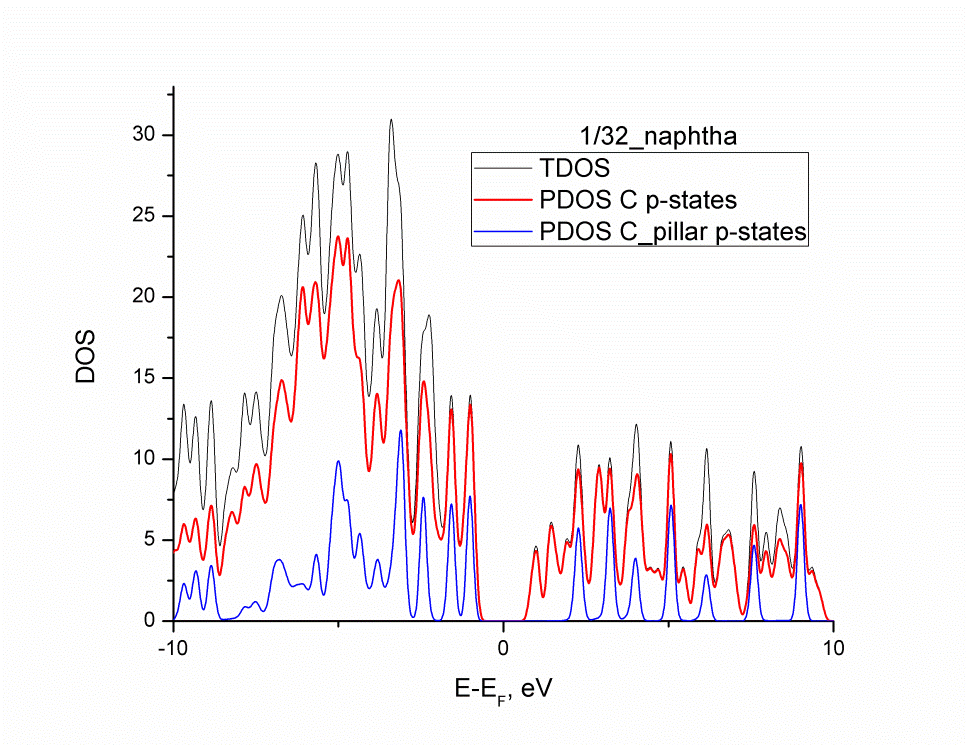


Figure S 21. PDOS plot for MPG_1/32_naphtha.


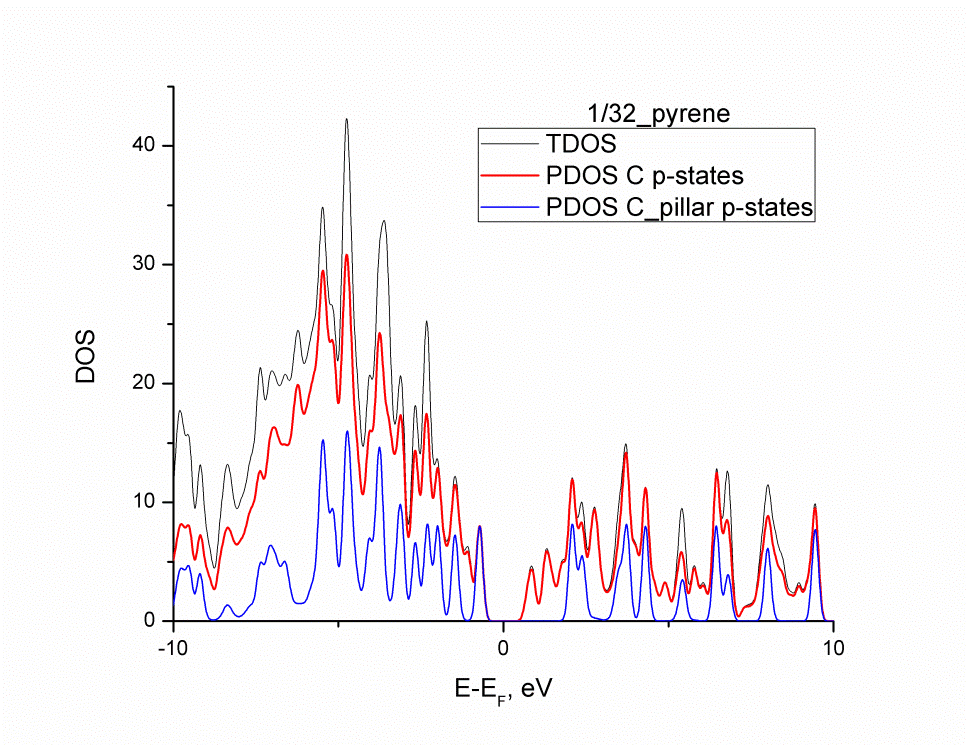


Figure S 22. PDOS plot for MPG_1/32_pyrene.

1. **Non isotropic pillar distribution in the adjacent interspaces of MPG materials.**

The effect in the band structure of the materials due to the non isotropic distribution of the pillars in the interlayer spaces of the system was further explored by considering different pillar distributions in the adjacent interlayer spaces of the phenyl derivatives for 1/18 and 1/32 pillar densities. Based on the optimized cells corresponding to these structures, we build new structures by relocating the pillars with respect to each other. Then, geometry optimization of the atom positions and the lattice parameters was done followed by the calculation of the band gap for each optimized structure according to the computational details that were used for the rest of the structures.

The relative distributions of the pillars in the interlayer spaces for 5 different configurations of **MPG_1/18_benz** can be seen in the following figure S23. MPG_1/18_benz_D1 corresponds to the original MPG_1/18_benz structure. We must mention that we could not find an *isotropic* distribution for 1/18 pillar density due to the size of the periodic model of the graphene with the specific number of C atoms. Each figure corresponds to 2x2x1 supercell in order to be easier to see the distribution of the pillars in both sides of the graphene layer. The position of the pillar above the graphene plane was kept constant, changing only the position of the pillar below the plane.


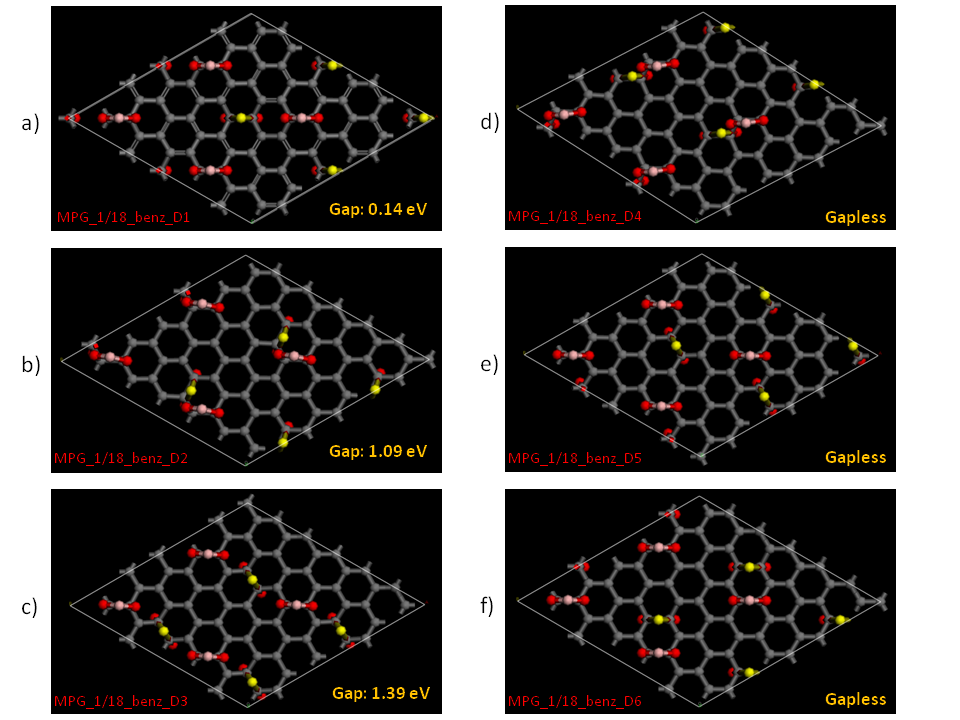


Figure S 23. a)-f). 2x2x1 supercell representations of the MPG_1/18_benz materials showing the redistributed positions of the pillars. MPG_1/18_benz_D1 corresponds to the initial structure considered in this study. Phenyl rings have been omitted for clarity. Yellow spheres correspond to the indicators of the position of the pillars in the underneath interlayer space.

We did not notice any change in the lattice parameters of the structures with the redistributed pillars after the optimization with respect to the initial structures. Calculation of the corresponding band gaps for all the configurations showed that both the relative distribution of the pillars and the relative orientation of the upper with the underneath pillars (D2, D3 and D5 configurations) can alter the electronic behavior of the material from semiconducting to semi-metal. The values of the band gap ranges from 1.39 eV (for D3 distribution) to gapless.

We followed a similar procedure and we build four different configurations for **MPG_1/32_benz**, which can be seen in Figure S24. Also for 1/32 structures, no changes in the lattice parameters of the structures with the redistributed pillars with respect to the initial structures were found after optimization. MPG_1/32_benz_D1 corresponds to initially considered structure which is also isotropic with respect to the distribution of the pillar in the two spaces. The isotropic distribution in D1 configuration correspond the largest band gap that was found (0.98 eV), where for the non isotropic configurations (D2 to D5) the band gap vary from 0.5 eV to nearly gapless.


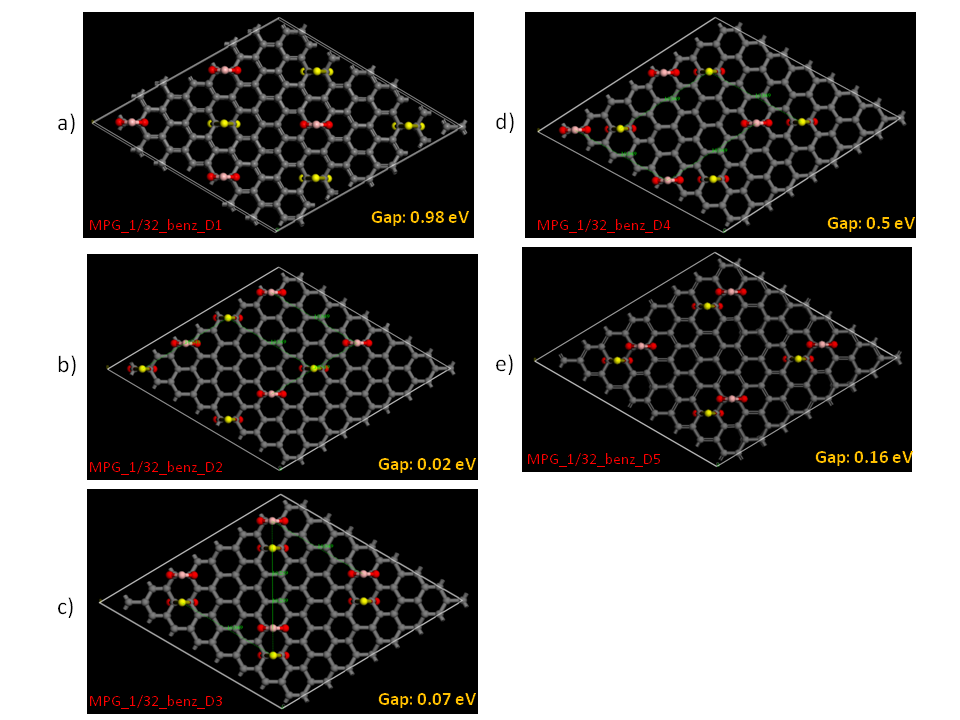


Figure S 24. 2x2x1 supercell representations of the MPG_1/32_benz materials showing the redistributed positions of the pillars. MPG_1/32_benz_D1 corresponds to the initial structure considered in this study which corresponds to the isotropic distribution of the pillars. Phenyl rings have been omitted for clarity. Yellow spheres correspond to the indicators of the position of the pillars in the underneath interlayer space.

1. **Computational details for the calculations on functionalized graphene monolayers.**

In order to further investigate the origin of the effect on the band gap in MPG materials, we build periodic models consisting of only one functionalized graphene layer and we calculated the corresponding band gaps. The models were built for the phenyl and pyrene derivatives and the three pillar densities considered in this study. The unit cell was increased to 50Å in the direction vertical to the plane of the graphene in order to avoid any interlayer interactions. A representative illustration of the periodic model can be seen in the following figure, which corresponds to the 1/8 pillar density for the phenyl derivative (MPG_1/8_benz).


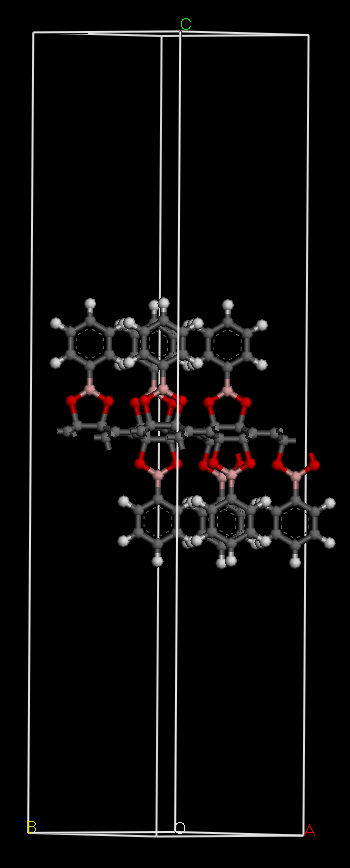


Figure S 25. 2x2x1 supercell of the periodic model that was build corresponding to the phenyl derivative of the graphene monolayer for 1/8 pillar density.

The periodic models were optimized and then the corresponding electronic properties were calculated. The calculated band gaps for the monolayers are similar to those corresponding to the 3D analogs. For the 1/18 and 1/32 pillar densities, identical values were found where for the 1/8 density a deviation of the order of 0.1 eV was found with respect to the 3D structures. It can be deduced that the linkage does not affect the values of the band gap. Nevertheless, the linkage still consist an important feature of the material with direct implications on the mechanical properties and in the engineering of the interlayer space between the graphene layers.

1. **Calculations on the flexibility of MPG’s.**

In order to explore the mechanical properties of the structures we defined the bulk and shear moduli of the structures. To do so, we first optimized the crystal structures using Dreiding force field^[[1]](#endnote-2)^. All calculations were made with the Forcite module included in the Materials Studio V2.2 Package. The optimized structures were then used to calculate the bulk modulus and shear modulus in order to estimate the flexibility of the structures. Constant strain method was used applying a given strain a series of steps. Maximum strain amplitude was set to be 0.003. Structures were allowed to be optimized after each step. The calculation of the two modulis was made according to Voigt formulation.

This procedure was done for all the MPG structures that were considered in this study. Two more MPG structures were also considered corresponding to MPG_1/128_benz and MPG_1/128_triphen. The same modulis were also calculated for diamond, the crystal silicon and the graphite structures in order to compare their flexibility with the MPG structures. The results are shown in Table S2. It can be seen that the new structures show considerably increased flexibility in comparison with the test structures. Moreover, the flexibility of the new structures increases as we add longer pillars between the graphene layers or by decreasing the pillar density per square area of graphene area MPG’s.

**Table S2:** Bulk and shear moduli for the various MPG structures examined in this study.

| name | bulk modulus (GPa) | shear modulus (GPa) |
| --- | --- | --- |
| diamond | 664 | 442 |
| silicon | 487 | 281 |
| graphite | 363 | 227 |
| MPG_1/8_benz | 160 | 70 |
| MPG_1/8_biphen | 139 | 57 |
| MPG_1/8_triphen | 122 | 50 |
| MPG_1/8_naphtha | 153 | 68 |
| MPG_1/8_pyrene | 162 | 81 |
| MPG_1/18_benz | 118 | 59 |
| MPG_1/18_biphen | 89 | 44 |
| MPG_1/18_triphen | 73 | 36 |
| MPG_1/18_naphtha | 102 | 51 |
| MPG_1/18_pyrene | 96 | 45 |
| MPG_1/32_benz | 126 | 64 |
| MPG_1/32_biphen | 83 | 41 |
| MPG_1/32_triphen | 67 | 33 |
| MPG_1/32_naphtha | 96 | 48 |
| MPG_1/32_pyrene | 87 | 42 |
| MPG_1/128_benz | 103 | 58 |
| MPG_1/128_triphen | 60 | 32 |

1. S. L. Mayo, B. D. Olafson, W. A. Goddard, *J. Phys. Chem*. 1990, **94**, 8897-8909. [↑](#endnote-ref-2)
